# A Cross-Species Study of Gesture and Its Role in Symbolic Development: Implications for the Gestural Theory of Language Evolution

**DOI:** 10.3389/fpsyg.2013.00160

**Published:** 2013-06-06

**Authors:** K. Gillespie-Lynch, P. M. Greenfield, Y. Feng, S. Savage-Rumbaugh, H. Lyn

**Affiliations:** ^1^Department of Psychology, University of CaliforniaLos Angeles, CA, USA; ^2^Great Ape TrustDes Moines, IA, USA; ^3^Department of Psychology, University of Southern MississippiLong Beach, MS, USA

**Keywords:** gestural theory of language evolution, language-enculturated apes, symbolic development, cross-species comparisons, gesture, communication development, language development

## Abstract

Using a naturalistic video database, we examined whether gestures scaffold the symbolic development of a language-enculturated chimpanzee, a language-enculturated bonobo, and a human child during the second year of life. These three species constitute a complete clade: species possessing a common immediate ancestor. A basic finding was the functional and formal similarity of many gestures between chimpanzee, bonobo, and human child. The child’s symbols were spoken words; the apes’ symbols were lexigrams – non-iconic visual signifiers. A developmental pattern in which gestural representation of a referent preceded symbolic representation of the same referent appeared in all three species (but was statistically significant only for the child). Nonetheless, across species, the ratio of symbol to gesture increased significantly with age. But even though their symbol production increased, the apes continued to communicate more frequently by gesture than by symbol. In contrast, by 15–18 months of age, the child used symbols more frequently than gestures. This ontogenetic sequence from gesture to symbol, present across the clade but more pronounced in child than ape, provides support for the role of gesture in language evolution. In all three species, the overwhelming majority of gestures were communicative (i.e., paired with eye contact, vocalization, and/or persistence). However, vocalization was rare for the apes, but accompanied the majority of the child’s communicative gestures. This species difference suggests the co-evolution of speech and gesture after the evolutionary divergence of the hominid line. Multimodal expressions of communicative intent (e.g., vocalization plus persistence) were normative for the child, but less common for the apes. This species difference suggests that multimodal expression of communicative intent was also strengthened after hominids diverged from apes.

## Introduction

The idea that language evolved from a primarily gestural mode of communication is centuries old (Condillac, 1746; Hewes, 1973, 1976; Corballis, 2002, 2009). Evidence that may support a gestural origin of language includes the relatively early emergence of bipedalism (freeing up the hands to gesture), the possibility that modern hand configurations arose much earlier than the modern vocal tract, the variability, and flexibility of non-human primates’ gestural abilities relative to their vocal communication, evidence of shared neural substrates for manual action and language, and the finding that chimpanzees exhibit enhanced laterality in communicative gesture relative to other types of action (Greenfield, 1991, 2008; Lieberman, 1998; Rizzolatti and Arbib, 1998; Corballis, 2002; Hopkins et al., 2005; Molnar-Szakacs et al., 2006; Armstrong and Wilcox, 2007; Armstrong, 2008).

Because behaviors such as language and gesture do not fossilize, evolutionary links between gesture and language are impossible to prove. However, there is strong evidence in favor of an ontogenetic relationship between gesture and language. Gestures may allow infants to refer to objects before mastering their names and to gain input about relations between words and objects (see Iverson and Goldin-Meadow, 2005 for a discussion of this). Deictic gestures (referring to an object or location) allow reference to grow from the immediate context toward abstraction by helping infants understand the link between symbols and referents (Werner and Kaplan, 1984/1963; Zukow-Goldring, 1996; de Villiers Rader and Zukow-Goldring, 2010). Deictic gestures are more common than words early in development and predict linguistic development in both typical and atypical human populations across many cultures (Bates et al., 1975; Caselli, 1983; Caselli and Volterra, 1990; Goldin-Meadow and Morford, 1994; McGregor and Capone, 2001; Iverson and Goldin-Meadow, 2005; Rowe et al., 2008).

Children typically begin using gestures several months before they begin using words (Goldin-Meadow and Morford, 1994). Indeed, words typically become more common than gestures within the second year of life (Iverson et al., 1994; Iverson and Goldin-Meadow, 2005; Pizzuto and Capobianco, 2007). Even as gestures decline in importance in one-element communications, they remain important as part of two-element combinations (Greenfield and Smith, 1976; Capirci and Volterra, 2008). Gesture-symbol combinations precede the development of symbol-symbol combinations in both language-enculturated apes and human children (Iverson and Goldin-Meadow, 2005; Greenfield et al., 2008). Thus, gestures seem to provide a foundation for each new stage in early linguistic development.

Focusing on objects that were referred to in one modality before appearing in another modality at a later observation, Iverson and Goldin-Meadow (2005) found that 10–24-month old infants first referred to objects with communicative gestures before speech more often than with speech before gesture. Our goal in the present study was to see if this gestural scaffolding of specific vocabulary items would hold across the clade of human, chimpanzee, and bonobo – under similar conditions of a language-enriched environment. The method of Iverson and Goldin-Meadow (2005) was highly suitable for our purpose: to examine the role of gesture in the ontogeny of symbol use across the clade.

### Ontogeny and phylogeny in early communication development

More generally, we hypothesized that all three species would exhibit a shift from greater reliance on gestures to greater reliance on symbolic communication with development. Such evidence would support the gestural theory of the evolution of language. Because evolution is a series of ontogenetic sequences, with earlier stages, more preserved in evolutionary history than later ones, cross-species similarities in early developmental sequences provide relevant evidence for reconstructing phylogenetic history (Parker and McKinney, 1999).

The logic of cladistic analysis is such that traits found across an entire clade (defined as species with a common immediate ancestor) are likely to have existed in the common ancestor (Parker and McKinney, 1999). Hence, another basic type of evidence for the gestural theory of language evolution would be similarities in the function and form of gestures across the clade. Using video data, we therefore describe and compare the different types of gesture in bonobo, chimpanzee, and human at comparable stages of communicative development. This is the first time such data has been available to compare the role of gesture in communicative development across the clade.

### Comparing gesture across species

One of the primary challenges in comparing gestures across species is that definitions of gesture vary across studies. Gesture has been defined as specifically as communicative movements of the hands and as broadly as any visible bodily action (Kendon, 2000, 2004; Wilkins, 2003; Crais et al., 2004; Liebal et al., 2004; Müller, 2007; Pika, 2008). In the present study, we were open to communicative gesture involving body parts other than the hand. In addition, we operationalized communicative intention as separate from the gestural action itself. Perhaps most important, we utilized the same operational definition of gesture across all three species, a major methodological advance for the comparative study of gesture.

#### Defining communicative intention

Varying definitions of communicative intention, or evidence that a gesture was emitted in order to influence another, also complicate cross-species comparisons of gestures. Communicative intention is often indexed by the presence of attention-getting behaviors (such as vocalization), monitoring the attentional state of the addressee (e.g., gaze alternation between addressee and referent), or persistence in maintaining a gesture until a response is elicited (Bates et al., 1975; Bard, 1992; Krause and Fouts, 1997; Leavens and Hopkins, 1999).

Captive bonobos, chimpanzees, gorillas, and orangutans display clear evidence of communicative intention, or monitoring the attentional states of others, while gesturing. For example, they more frequently use purely visual gestures when their audience is facing them and communicate with vocalizations more when their audience is facing away (Tomasello et al., 1997b; Hostetter et al., 2001; Pika et al., 2005; Liebal et al., 2006; Genty et al., 2009). Two signing chimpanzees acquired the attention of their caregivers before gesturing and also exhibited gaze alternation between addressee and referent while gesturing (Krause and Fouts, 1997). Captive adult chimpanzees and orangutans also engage in gestural persistence when their communicative needs are not met (Leavens et al., 2005; Cartmill and Byrne, 2007). Thus, both non-language-enculturated apes in captivity and language-enculturated apes show evidence of communicative intent by monitoring the attentional state of others.

Although many studies of human development use eye contact to infer that a gesture is communicative, the majority of gestures produced by humans between 12 and 21 months of age may not co-occur with eye contact (Blake et al., 1992). Tactile contact may serve the same function as visual monitoring (Leung and Rheingold, 1981). Thus, some researchers of human gestural development require only that a gesture be directed toward another for it to be considered communicative, rather than specifying eye contact as a criterion (Crais et al., 2004). In order to be consistent with previous work examining ape gestures, we defined communicative intent for the purposes of the analyses reported in this paper in terms of persistence, attention eliciting behaviors (e.g., vocalization), or monitoring behaviors (e.g., eye contact) (Leavens and Hopkins, 1999).

Previous research comparing human infants to captive apes (at a mean age of 18 years) and language-enculturated adult apes revealed that apes exhibit more eye contact when gesturing than human infants do (see Leavens and Hopkins, 1999 for a review). In contrast, both captive and language-enculturated apes pair gestures with vocalizations less frequently than do human infants (see Leavens and Hopkins, 1999 for a review). Based on these findings, we hypothesized that the bonobo and chimpanzee would more frequently use eye contact to indicate communicative intent, whereas the child would more frequently use vocalizations to do so. This difference could be a key to the evolution of vocal language in humans, but not apes, after the split between *Homo* and *Pan* five to six million years ago.

#### Types of gestures

Unlike pre-linguistic children, apes who are not language-enculturated produce primarily dyadic (referring to the recipient of the gesture) rather than triadic (indicating a third entity) gestures (Camaioni, 1997; Pika et al., 2005; Pika, 2008). However, pointing to objects has been observed among captive apes (Leavens and Hopkins, 1999), language-enculturated apes (Miles, 1990; Call and Tomasello, 1994; Brakke and Savage-Rumbaugh, 1996; Krause and Fouts, 1997; Leavens and Hopkins, 1999; Tanner et al., 2006), and apes in the wild (Inoue-Nakamura and Matsuzawa, 1997; Vea and Sabater-Pi, 1998). Pointing with the index finger is relatively infrequently observed among captive or wild apes who have not been language-enculturated, but whole hand pointing (or indicative reaching) is commonly demonstrated by captive apes when they have a receptive audience (Call and Tomasello, 1994; Leavens and Hopkins, 1999).

Language-enculturated adult apes may exhibit more pointing with the index finger relative to reaching gestures than both captive apes (at a mean age of 18 years) and human infants younger than 19 months of age (Leavens and Hopkins, 1999). Thus, being raised in a linguistically enriched environment may facilitate the emergence of pointing across ape species (Miles, 1990; Brakke and Savage-Rumbaugh, 1996; Krause and Fouts, 1997; Tanner et al., 2006). Indeed, a language-trained orangutan exhibited more flexible pointing and better understanding of human points than a captive orangutan who had been trained to point (Call and Tomasello, 1994; Tomasello et al., 1997a). An emergent relationship between symbol training and pointing has even been observed among dolphins. Dolphins trained for 6 months to communicate with an underwater symbol communication board spontaneously began to exhibit pointing behaviors with associated gaze monitoring in the presence of human trainers (Xitco et al., 2001). And two dolphins who had been in a language-training program for over 15 years both showed complex understanding of human pointing behaviors (Herman et al., 1999; Pack and Herman, 2007). These findings indicate both the importance of the social environment for the emergence of communicative potential and the developmental connection between symbolic and gestural communication.

However, pointing increases with age for human infants (Locke et al., 1990; Franco and Butterworth, 1996; Masataka, 2007). Because pointing could also increase over age for language-enculturated apes, it is important to compare language-enculturated apes and humans when both are at early stages of development, as we do in the present study. Indeed, it is quite possible that pointing occurs less frequently for younger apes than for older ones, as is the case for children.

There is also evidence that young language-enculturated apes use their lexigrams (non-iconic visual signifiers) to request more often than to indicate (Greenfield and Savage-Rumbaugh, 1990; Lyn et al., 2011). Reaching is a gesture that often signifies request, whereas pointing is a gesture that often signifies indication. Young children do not show an increase in the frequency of communicative reaching over the second year of life (Franco and Butterworth, 1996). Given all of these facts, one might expect that the child would point relatively more than the young apes and reach relatively less.

Tomasello (2006) theorizes that apes, unlike year-old children, are not motivated to share experience with others for its own sake. Of all gestures, the gesture of holding up an object to show another is perhaps the most unambiguous example of social sharing for its own sake. In support of his point, Tomasello notes that, around 12 months of age, infants hold up objects to show to others, whereas apes do not. However, a comparative study of showing gestures in a chimpanzee, bonobo, and child is required to empirically confirm or disconfirm this assertion; the present study fills this gap.

## Materials and Methods

### Participants

A chimpanzee, a bonobo, and a human child participated in the current study. The ape participants were Panpanzee, a female chimpanzee (*Pan troglodytes*), and Panbanisha, a female bonobo (*Pan paniscus*), who was 6 weeks older than Panpanzee. These apes were reared together at the Language Research Center in Atlanta, Georgia in a language enriched environment where they learned to communicate with their caregivers using gestures, vocalizations, and lexigrams (arbitrary visual symbols representing words). The language enriched environment included ongoing activities wherein caregivers and apes communicated through gestures, lexigrams, and vocalizations, as well as daily language-testing sessions (Savage-Rumbaugh et al., 1993). The caregivers also communicated *via* English speech. As with human children, lexigram symbols were learned within the context of ongoing activities that were relevant to the apes (Savage-Rumbaugh et al., 1993, 1998; Brakke and Savage-Rumbaugh, 1996).

Inter-individual routines consisted of play and exploration both within the apes’ living space and while foraging through the forest outside their home. While the same lexigrams were available both inside and outdoors, the lexigram boards used while exploring the woods were plastic covered printed sheets, while the keyboards inside were electronic. When a lexigram on one of these inside boards was pressed, an electronic voice spoke the word that lexigram represented. Lexigram boards used during exploration were designed to be highly portable and did not emit words when pressed. In order to capture all possible communication on video, caregivers spoke the word for each lexigram touched on the more portable lexigram boards. The apes understand human speech and often respond to a caregiver’s speech through lexigrams and/or gestures (Savage-Rumbaugh et al., 1993).

The human participant in this study was a typically developing girl, GN, who was reared by her middle-class parents in a typical European-American linguistic environment. The observations were done at home in naturally occurring situations, usually, but not always, indoors.

### Data sources

Video data of the bonobo, Panbanisha, and the chimpanzee, Panpanzee, were recorded from soon after birth until Panpanzee was moved to a new location when she was 3 years and 11 months of age. Biweekly or monthly recordings of varying length were conducted until the apes were 26 months of age; subsequent recordings occurred every few months. Monthly hour-long videos of the child, GN, were recorded from 8.5 months of age till almost 2 years of age. In a few instances, it was necessary to return a second day to complete the hour for a particular month. In each case, the video was naturalistic; there was no attempt to make the settings across species more similar than they actually were.

Following the methods of Iverson and Goldin-Meadow (2005) as closely as possible, we wished to examine communication between the onset of one element and two-element symbol production. Based on the communicative level observed in the recordings and the availability of usable data, we selected approximately two half-hour sessions each month for the chimpanzee and bonobo from 12 to 26 months of age and an hour per month from 11 to 18 months of age for the child. After accounting for variations in the quantity of usable data available for each participant across the specified age range, approximately 14–15 h of video were coded for the bonobo and chimpanzee respectively while 8 h of video were coded for the child.

While the bonobo, Panbanisha, first used lexigrams communicatively at 11 months of age, the chimpanzee, Panpanzee, began lexigram use at 13 months of age (Brakke and Savage-Rumbaugh, 1996). However, usable video data was only available for both apes beginning at 12 months of age. We identified the onset of one-word speech for the child, GN, by viewing backwards from 12 months of age until a video was identified wherein the child did not utter a word, at 10 months of age. Thus, the onset of data analysis for each participant was the following: bonobo at 12 months of age, chimpanzee at 12 months of age, and the human child at 11 months of age.

As in Iverson and Golden-Meadow’s study, the offset of data analyses for the child coincided with clear evidence of multiword speech operationalized as five occurrences of different word combinations (18 months). Because the apes combined lexigrams less frequently than the child combined words and continued to use mainly single words throughout the study, the offset of ape data analysis was determined by the availability of usable data. After 26 months of age, no videos were available of the chimpanzee, Panpanzee, until she was 30 months of age. Thus, data analysis was terminated at 26 months of age for both apes. Generally, videos focused on only one ape at a time. When videos included both apes engaging in activities with one another, the video could be coded for either ape as long as the ape was visible for the majority of the sampled video. GN’s data captured daily interactions at home and in her backyard in various contexts (such as eating breakfast, celebrating a birthday, playing with dolls, etc.) to give a reflection of normal daily interactions with her parents and other people. The environment in which the child was filmed more closely approximated the environment of participants in the Iverson and Goldin-Meadow (2005) study than did the apes’ environment, in that the apes spent a far greater proportion of their time exploring a large outdoor area with a much larger number of possible referents than are available in a home.

### Gesture coding schemes

Coding schemes for both apes and the child were developed based on methods developed by Iverson and Goldin-Meadow (2005). Two types of communication were coded: (1) gestures and (2) lexigrams or speech.

**Figure 1:**
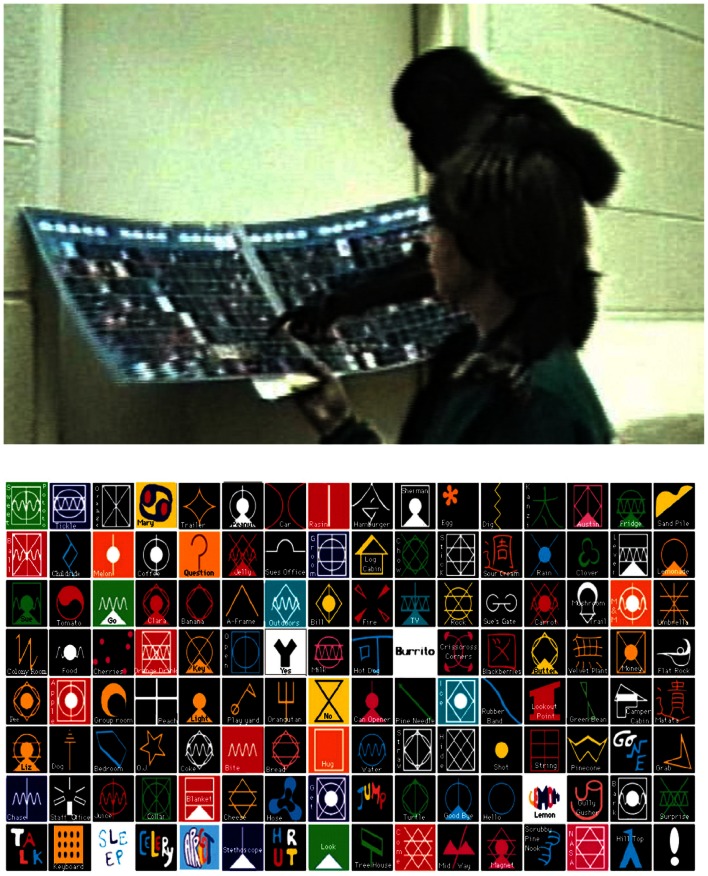
**An example of a *lexigram* use (top) and an image of lexigram board (bottom)**. *Lexigram* use was defined as touching a lexigram while the referent was glossed by caregiver or electronic voice on lexigram board.

Lexigram use (for the apes) was defined as touching a lexigram while the referent was glossed by caregiver or electronic voice on the lexigram board (see Figure 1). Speech (for the child) was coded only if directly interpreted or responded to by a caregiver to make coding of speech as similar as possible to coding of lexigrams.

**Figure 2:**
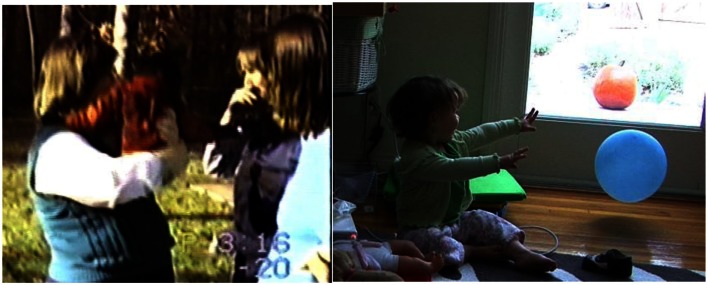
**Examples of a *reach* gesture by an ape and the human**. *Reaches* involve actively extending a limb toward a referent without contacting it. Left: ape example – Sue, Panbanisha’s primary attachment figure, has been holding Panbanisha. Someone new (Linda) wants to hold her. Linda takes Panbanisha (1 year, 9 days) who reaches for Sue in this frame. Linda walks away with Panbanisha who vocalizes loudly in protest. Right: human example – Dad throws balloon; GN (15 months, 26 days) vocalizes and reaches toward it. She turns toward videographer, then dad. “You can go get it,” he says; and she does.

**Figure 3:**
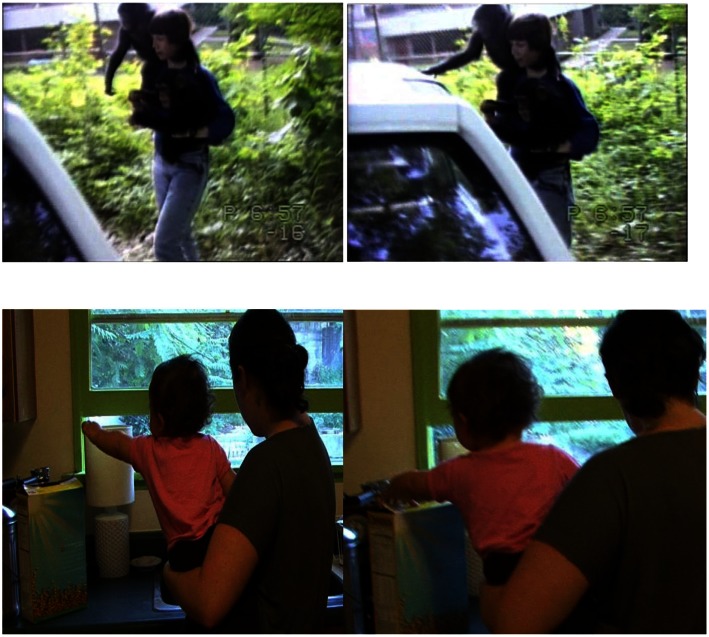
**Examples of a *reach-touch* gesture sequence (*reach* on left and *touch* on right of each pair)**. *Reach-touch* is the same as *reach* except that contact is made with the referent, but only after a response from the caregiver. Top: ape example-Carrying Panbanisha and Panpanzee, Sue says, “Tell us where you wanna go.” Panpanzee guides Sue by taking her hand. Then as they near car, Panbanisha (17 months, 26 days) gestures to it and Sue walks toward it till they can touch it. “Oh you wanted to go in Steve’s car,” she says, and they peer inside. Bottom: human example – Mom is holding GN (11 months, 7 days) and washing something in the sink. GN reaches toward Cheerios. Mom walks closer so GN can reach into box and get Cheerios.

Gestures were coded according to their form into one of the following categories: *reach*, *reach-touch*, *point*, *point-touch*, *up*, *head-point*. *Other gestures* exhibited by only one species will be discussed in the next paragraph. *Reach* involved actively extending a limb without contacting a referent (see Figure 2). *Reach-touch* was the same as *reach* except that contact was made with the referent, but only *after* a response from the caregiver (see Figure 3). We required that the caregiver respond before the ape contacted the object in order to distinguish between reaches that were direct actions upon an object and reaches that were gestures requiring a response from another in order to allow action upon an object.

**Figure 4:**
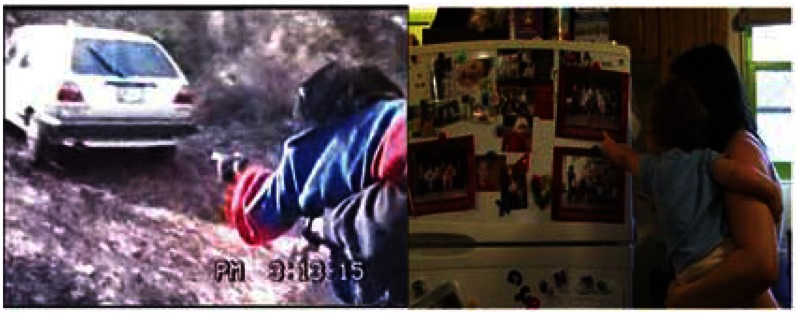
**Examples of a *point* gesture**. *Point* involves extending an arm with the index finger extended toward an object without touching it. Left: ape example – The caregiver asks Panpanzee (22 months, 20 days) where she wants to go and she points toward the car. They walk toward the car. Right: human example – GN (13 months, 9 days) points at picture on fridge while vocalizing. “What do you see?” Mom asks. “There’s GD in a picture,” Mom continues. GN points again (not shown) and then turns to point at actual GD, her older brother, who is out of view.

**Figure 5:**
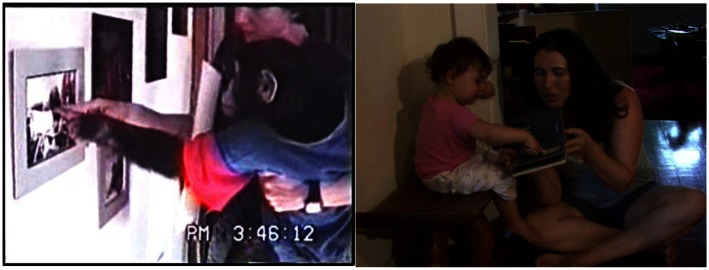
**An example of a *point-touch* gesture by an ape and a human**. *Point-touch* is a point wherein the participant ends the point by touching the referent *without* moving her finger along it or manipulating it. Left: ape example – Sue, the caregiver, and Panpanzee were walking around Sue’s house, and Panpanzee pointed toward the picture. They walked to it, and Panpanzee (22 months, 20 days) point-touched it. Sue then also point-touched it and commented on the picture. Right: human example – Mom is reading book to GN (12 months, 6 days). GN gestures toward it, then point-touches moon in it. “Look, there’s the moon,” says Mom.

**Figure 6:**
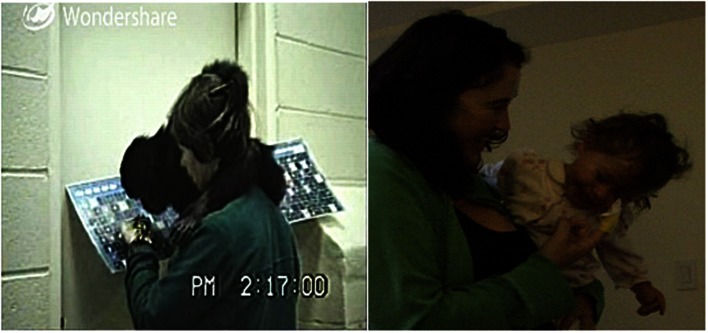
**Examples of a *head-point* gesture by an ape and a human**. *Head-point* involves indicating an object by using one’s head. Left – Ape example: Sue shows Panbanisha (22 months, 27 days) a chain of keys and asks her to pick a key to open the door. Panbanisha touches the keys with her face/head. Sue opens the door. Right – human example: mom holds up finger puppet, saying “See it’s a baby.” She pretends to give it a bottle. GN (14 months, 1 day) laughs and head-points it. “Yeah it’s a baby,” Mom says.

**Figure 7:**
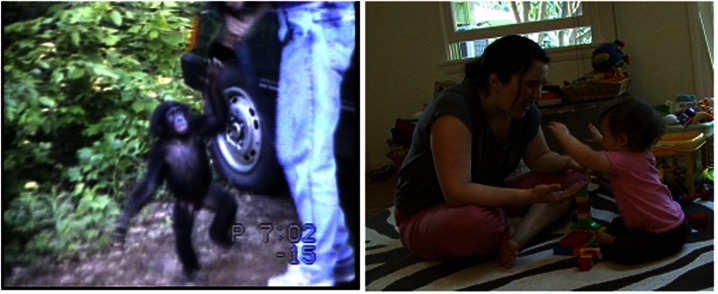
**Examples of an *up* gesture**. *Up* involves raising the arm/arms above the head with the implied intention of being picked up. Left: ape example – Panbanisha climbing on car. Sue, holding Panpanzee on shoulders, says “Panban, don’t do that.” Panbanisha (17 months, 26 days) gets down and comes to Sue with arm raised for *up*. Sue puts Panpanzee down. Panpanzee briefly shoves Panbanisha and scampers off. Then Sue picks Panbanisha up. Right: human example – GN and Mom are playing with Lego blocks. GN (11 months, 7 days) raises arms up. Mom helps her up.

*Point* involved extending an arm with the index finger extended toward an object without touching it (see Figure 4). *Point-touch* began as a point but the participant ended the point by touching the referent without moving her finger along it or manipulating it (see Figure 5). Unlike *reach-touch*, we did not require a caregiver response in order for *point-touch* to be considered a gesture because *point-touches* often occurred in relation to objects that the child or ape could touch without a caregiver response. We distinguished between *point-touches* as gestures and touching something with the index finger as an exploratory action by requiring that *point-touches* not involve manipulation of the object. *Head-point*, indicating an object by using one’s head (see Figure 6), was observed once from the child and once from the bonobo. *Up* involved raising the arm/arms above the head with the implied intention of being picked up (see Figure 7).

Other gestures were exhibited by the child, but not the apes; these included *show*, *head shake*, *nod*, *open*, *wave*, and *shhh*. *Show* involved holding an object into the line of gaze of another while looking toward the person’s face without subsequently giving the object to the other. *Head shake* involved shaking the head from side to side. *Nod* involved moving the head up and down. *Open* involved moving a partially curled hand back and forth while reaching toward a door knob. *Wave* involved moving an open hand back and forth while looking at another person. *Shhh* involved holding a finger to pursed lips.

Still others were exhibited only by the chimpanzee. Only the chimpanzee was observed to once exhibit a *give* gesture, or outstretched palm without attempting to grab an object. The chimpanzee was also the only participant to use *guide hand* gestures wherein she moved the caregiver’s hand into a reaching position. The bonobo was alone in exhibiting no unique gestures.

**Figure 8:**
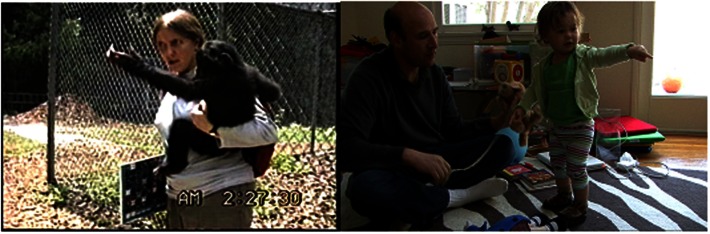
**Examples of a *go* gesture by an ape and a human**. *Go* involves reaching and pointing when no referent is visible (even when the camera pans to give clear view of scene). Left: ape example – Rose is standing near a fence holding Panpanzee (21 months, 2 days). Panpanzee gestures to go. Rose walks in the direction gestured. Right: human example – Dad asks GN (15 months, 26 days) if she wants pasta. She says no and points go. He stands up and says “Let’s go.”

When gestures were deictic, they were also assigned a likely referent. Two clues to reference were used: the caregiver’s behavioral or verbal response to the gesture and the object or person which the gesture pointed toward. Gestures that involved reaching or pointing into the distance with no visible referent with the likely intention of causing motion in the indicated direction were interpreted as meaning *go* (see Figure 8). Gestures that involved reaching or pointing toward the ground with the likely intention of being lowered to the ground were interpreted as meaning *down*.

Each gesture and lexigram was also coded as either communicative or non-communicative. Communicative gestures or lexigrams possessed at least one of the following properties: persistence, eye contact, or vocalization (note: vocalization is different from speech). Persistence involved repeating a gesture or lexigram use at least two times in a row, going out of one’s way to communicate, or maintaining a communication until responded to. Eye contact involved turning the head toward or looking at a caregiver’s face immediately before, after, or during the gesture. Vocalization involved vocalizing at the same point in time as a communication or immediately prior to or after it.

We also recorded for each gesture and lexigram whether the behavior was an imitation of an immediately preceding behavior by the caregiver.

### Reliability of video coding

Inter-rater reliability was established by calculating the percentage agreement, or the frequency with which both coders made the same decision divided by the sum of agreements and disagreements, between two independent coders for the existence, type, and quality of gestures. This was a conservative measurement of reliability because agreement on all of the behaviors that were *not* instances of a given category were not taken into account. Inter-rater reliability for each ape and the child was established on 2 h and 30 min of video for each ape and 2 h and 40 min of video for the child across the age range sampled for the study. Percentage agreement was used as a reliability measurement in preference to correlation because all coding consisted of binary (presence-absence) judgments. Percentage agreement was used in preference to Cohen’s kappa because we did not count agreed upon absences, so there was no 2 × 2 matrix to analyze; such a matrix is required for the kappa statistic. Most likely these are the same reasons why percentage agreement was used in previous research in this topic area (e.g., Iverson et al., 1994; Iverson and Goldin-Meadow, 1997; Crais et al., 2004). See Table 1 for reliability rates for specific gestures and individuals.

**Table 1:** **Reliability: percentage agreement between two independent coders**.

	Panpanzee	Panbanisha	Human child
Gesture/symbol existence	83	81	80
Gesture/symbol type	80	82	98
Referent	94	87	95
Eye contact	89	90	83
Vocalization	100	97	93
Persistence	87	84	90
Communicativeness	81	81	97

*Agreement on communicativeness meant that both coders agreed that one of the following indices of communicativeness was associated with the gesture: persistence, eye contact, or vocalization*.

## Results

### Cross-species similarity in the form and function of gestures

**Figure 9:**
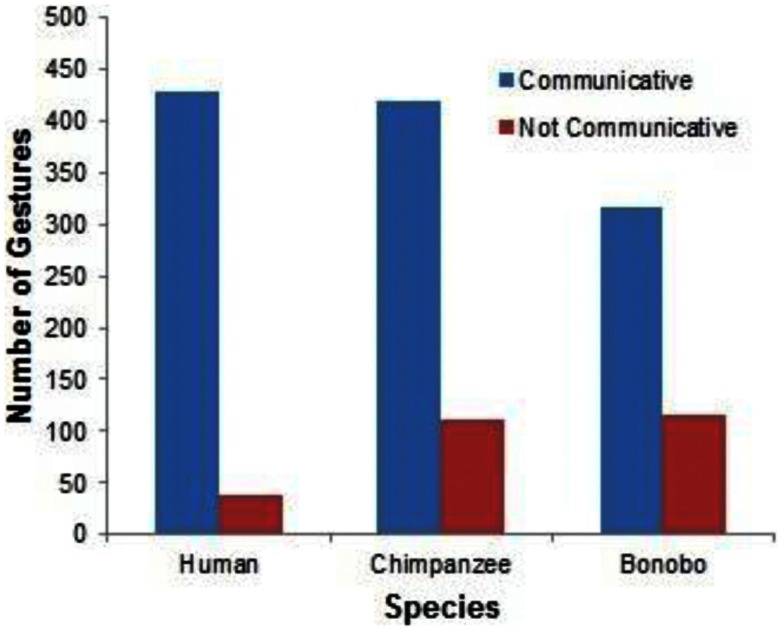
**A comparison of communicative (as defined by eye contact, vocalization, or persistence) relative to non-communicative gestures across species**.

The most basic finding, and one that is central to the gestural theory of language evolution, is the similarity of gestures among bonobo, chimpanzee, and human child at comparable periods of development (see examples in the video frames presented in Figures 2–8). The following analyses included only gestures that were not immediate imitations of caregiver behaviors. Binomial tests revealed that gestures were more frequently classified as communicative (that is, associated with eye contact, vocalization, or persistence) than non-communicative for the bonobo (*p* < 0.0001), the chimpanzee (*p* < 0.0001), and the human (*p* < 0.0001). See Figure 9 for the total number of communicative and non-communicative gestures for each species.

Following Iverson and Goldin-Meadow (2005), subsequent analyses focus on communicative gesture and symbol use, excluding from analysis behaviors that were not associated with eye contact, vocalization, or persistence.

### Modes of expressing communicative intent across the clade

**Figure 10:**
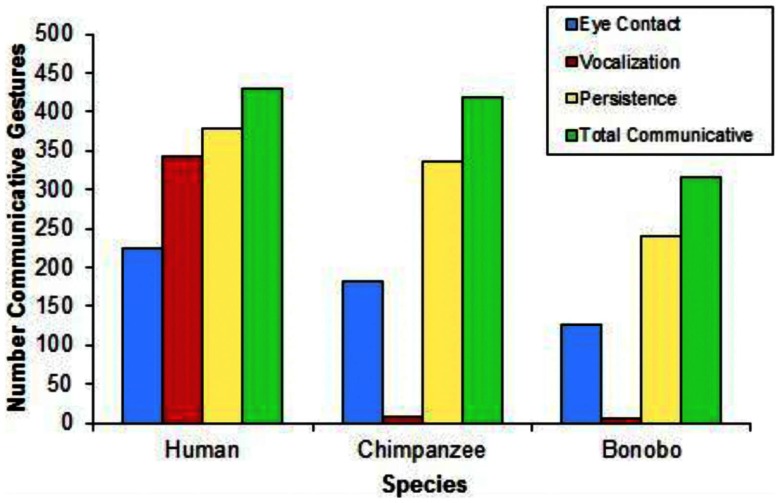
**A comparison of the use of eye contact, vocalization, or persistence when gesturing relative to total communicative gestures across species**.

Figure 10 presents the frequency with which gestures were paired with eye contact, vocalizations, and persistence across species. The child exhibited eye contact during 225, vocalization during 343, and persistence during 379 of 429 communicative gestures. The chimpanzee exhibited eye contact during 181, vocalization during 7, and persistence during 335 of 419 communicative gestures. The bonobo exhibited eye contact during 127, vocalization during 5, and persistence during 241 of 316 communicative gestures.

Three findings concerning the expression of communicative intent are of particular relevance to the evolution of language: one is that all three species use the complete array of behaviors that signal communicative intent: eye contact, vocalization, and persistence (Figure 10). The second is that the largest single difference between the human child and the apes is in the use of vocalization to signal communicative intent (Figure 10). As predicted, a higher proportion of the child’s communicative gestures were paired with vocalizations, compared with either the bonobo [χ^2^(1) = 445.853, *p* < 0.0001] or the chimpanzee [χ^2^(1) = 532.697, *p* < 0.0001], who did not differ from each other (*p* = 0.925). Contrary to predictions, a higher proportion of the child’s communicative gestures were paired with eye contact, compared with either the chimpanzee [χ^2^(1) = 6.901, *p* = 0.009] or the bonobo: χ^2^(1) = 10.483, *p* = 0.001, who did not differ from each other (*p* = 0.458).

The third important finding concerning the expression of communicative intent was not foreseen: the child used a much higher proportion of multimodal expressions of communicative intent than the apes did. 84% of the child’s communicative gestures utilized more than one means of signaling communicative intent; in contrast, only 23% of the chimpanzee’s communicative gestures and 22% of the bonobo’s communicative gestures utilized more than one means of signaling communicative intent [human vs. chimpanzee: χ^2^(1) = 314.901, *p* < 0.0001; human vs. bonobo: χ^2^(1) = 281.918, *p* < 0.0001; no difference between chimpanzee and bonobo, *p* = 0.877].

### Using gesture to scaffold lexical acquisition

We expected to find that gestures preceded symbol use more often than the reverse for the human child and the language-enculturated apes. Following Iverson and Goldin-Meadow (2005), we focused upon referents that were first referred to in one modality (gesture vs. symbol) in one session and later referred to in a different modality during a different session. We excluded from analysis referents first referred to by both a symbol and a gesture during the same observation session. For the child, 10 objects or actions were first referred to with gesture before speech while only one was first referred to with speech. A binomial test revealed that reference was more likely to occur first in gesture than in speech for the child (*p* = 0.012). Although a qualitatively similar pattern was observed for the language-enculturated apes, it was not statistically significant. The bonobo referred to five elements first through gesture and one element first with a lexigram (*p* = 0.219). The chimpanzee referred to three items first through gesture and one element first with a lexigram (*p* = 0.625). While the same qualitative pattern of symbols appearing first in gesture and only later in speech was observed across species, this pattern was statistically significant only for the child.

We also hypothesized that all three species would exhibit a shift from greater reliance on gestures to greater reliance on symbols (words for the child, lexigrams for the apes) with increasing age. In order to ensure that a varied range of contexts were represented when assessing patterns of communicative development, we compared the frequency of gesture and symbol use during the first half of the study to the frequency of gesture and symbol use during the second half of the study. Thus, we compared observations from the first 7 months of the study to observations from the last 7 months of the study for the apes and observations from the first 4 months of the study to observations from the last 4 months for the child. Because there were an uneven number of data points for the apes, data from their 19th month of age, the middle data point, was excluded from analysis. Analyses focus on frequency of use rather than the number of referents referred to within a given modality.

**Figure 11:**
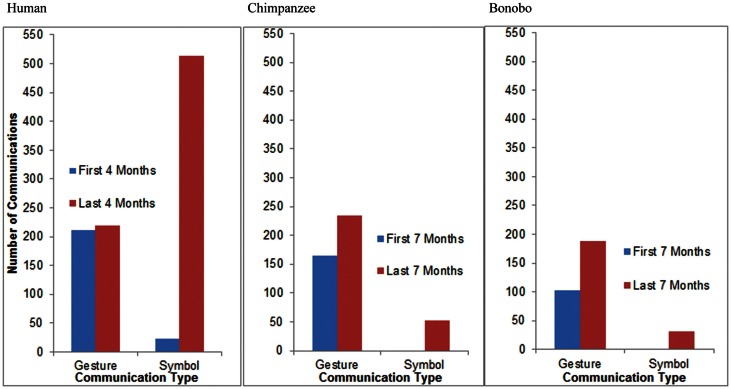
**A cross-species comparison of the frequency of communicative gestures relative to symbols during the first and the second half of the study**. (The bonobo produced two lexigrams in the first 7 months of the study, but, because of the necessary scale of the graphs to capture high frequency categories, they are not visible in the right-hand panel.)

Between 11 and 14 months of age, GN, the child, produced 211 communicative gestures and 23 words during observation sessions. Between 15 and 18 months of age, she produced an average of 219 communicative gestures and 513 words during observation sessions (Figure 11). A Fisher’s test revealed that the proportion of words relative to gestures increased significantly with age for the child (*p* < 0.0001). Between 12 and 18 months of age, Panpanzee, the chimpanzee, produced 165 communicative gestures and no lexigrams during observation sessions. Between 20 and 26 months of age, she produced 234 communicative gestures and 52 lexigrams during observation sessions (Figure 11). A Fisher’s test revealed that the proportion of symbols relative to gestures increased significantly with age for the chimpanzee (*p* < 0.0001). Between 12 and 18 months of age, Panbanisha, the bonobo, produced 103 communicative gestures and 2 lexigrams during observation sessions. Between 20 and 26 months of age she produced 188 communicative gestures and 32 lexigrams during observation sessions (see Figure 11.) A Fisher’s test revealed that the proportion of symbols relative to gestures increased significantly with age for the bonobo (*p* = 0.0002). Thus, increasing reliance on symbols relative to gestures was observed across the course of the study, regardless of species. From a common base of communicative gestures, all three species developed symbols.

### Reaching, pointing, and showing

Given that children indicate more, whereas language-enculturated apes request more on the symbolic level, we expected language-enculturated apes to exhibit a greater proportion of reaches relative to points when compared to a human toddler of a similar age. We focused upon canonical examples of communicative reaching and pointing and excluded from analysis head-points, point-touches, and reach-touches.

**Figure 12:**
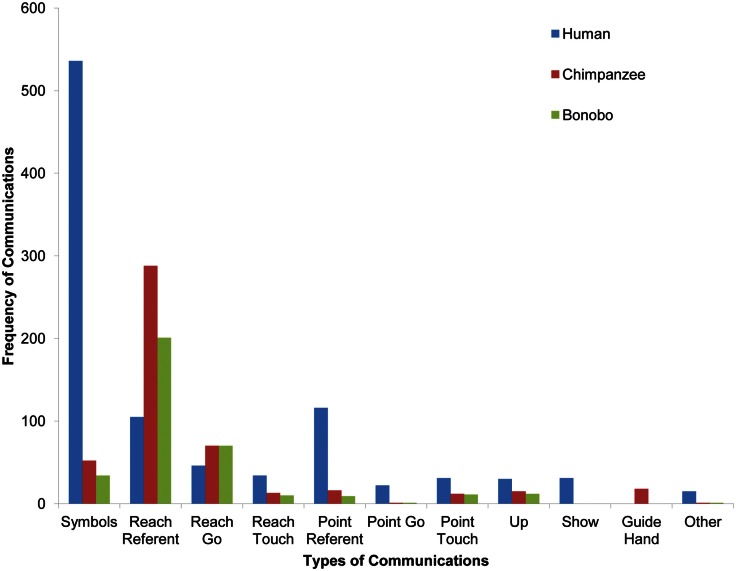
**A comparison of types of communicative gestures/symbols across species**. Head-point is classified as other due to its infrequency. (The chimpanzee and bonobo each produced one *point-go* and one *other* gesture, but because of the necessary scale of the graphs to capture high frequency categories, they are not visible.)

Visual inspection of the frequency of different communication types across the clade (depicted in Figure 12) suggests that the child used more symbols (as expected from the comparative developmental analysis in Figure 11) and pointing than the apes, whereas the apes used more reaching gestures. In order to capture the referential function of gestures, points and reaches used to indicate specific entities are depicted separately from points and reaches coded as “go” in Figure 12. However, because we did not have specific hypotheses about gestures depicting actions vs. entities, gestures with the same form are reported together in the following analyses irrespective of their referential function.

The child produced 138 points and 151 reaches over the course of the study. The bonobo produced 11 points and 271 reaches. The chimpanzee produced 17 points and 358 reaches. Fisher’s tests revealed that the child produced a higher proportion of points relative to reaches than the bonobo (*p* < 0.0001) and the chimpanzee (*p* < 0.0001). Given research suggesting that older language-enculturated apes exhibit more pointing than young infants and the overall increase in gestural frequency with age in the current study (Figure 11), the current findings suggest that language-enculturated apes may continue to develop the frequency with which they point after early childhood.

Exploring the idea that only human children are motivated to share experience for its own sake, we also compared the frequency of showing gestures in child, chimpanzee, and bonobo. In support of the idea that showing something to another may be uniquely human, the child was the only one to use a showing gesture – although the showing gesture was still not very frequent.

## Discussion

The current study, with its unique naturalistic video database for a young chimpanzee, bonobo, and human child, provides support for the role of gesture in language evolution. At the most basic level, we see a functional and formal similarity in gesture in all three species, with a young bonobo, chimpanzee, and child all at comparable periods of communicative development. Gestures served a communicative function across species in that they were usually paired with evidence of communicative intent. Similar types of gestures were also observed across species. According to the logic of cladistic analysis, these shared gestural capacities were likely present before the divergence of the three species five or six million years ago. Acknowledging the likelihood of gesture as a biological capacity in the clade’s common ancestor, it is nonetheless impossible to know to what extent and how it was actualized in behavior at that time. Still, given that human language, as we now know it, had not yet evolved at that time, this is one line of evidence for the gestural foundation of human language evolution.

### Gesture precedes symbols across species

The ontogenetic precedence of gesture before symbol across the clade provides another line of evidence for the gestural theory of language evolution. The frequency of symbol relative to gesture use increased with development across the clade. While phylogeny does not repeat ontogeny, it is the case that later stages of development cannot evolve without the ontogenetic foundation of earlier stages already being present (Parker and McKinney, 1999). Therefore, later stages of ontogenetic development tend also to evolve later.

### Reliance on gesture decreases for humans but not apes

However, there was also evidence of the phylogenetic divergence of humans and apes in the domain of communication. Symbols (in the form of words) became more frequent than gestures in the child’s later observations, whereas gestures remained more frequent than symbols (in the form of lexigrams) for the chimpanzee and bonobo throughout the study period. While the same qualitative pattern of reference being first achieved through gesture and only later through symbols was observed across the clade, this pattern was statistically significant only for the human participant. These developmental patterns are in line with the subsequent evolution of complex language in *Homo* but not *Pan*.

Like atypically developing humans communicating with typically developing humans, apes may face communicative barriers when trying to communicate with humans that they would not face when communicating with other apes. Findings from atypical human developmental trajectories suggest that developmental changes in gesture relative to symbol use may depend upon the match between individuals and communicative modalities. While most human children move from more gestures to more words with development, blind children do not exhibit this pattern (Iverson and Goldin-Meadow, 1997). Similarly, Caselli and Volterra (1990) found that between 10 and 11 months of age, both a hearing and a deaf toddler had equal numbers of words and gestures. Between 15 and 16 months, the speaking child’s gestural lexicon froze while the deaf child’s continued to expand.

Given limitations of the lexigram system devised to help apes communicate with us, compared with the flexibility of human speech, gestures may be a better match than symbols for apes but not humans. Unlike human speech, lexigram boards are not always available and have a constrained number of possible referents (a maximum of 256). Thus, a combination of gestures and symbols may confer some of the referential flexibility to language-enculturated apes that speech comes to provide to humans with development.

However, differences between the human and the apes in the observed frequency with which items transitioned from gesture to speech may also be attributable to the greater variety of contexts in which ape communication was observed relative to the human child; this greater variety of contexts greatly reduced the occurrence of the same referent across time, making the sample size too small to attain statistical significance. The human participant was assessed in a constant home environment, similar to that used by Iverson and Goldin-Meadow (2005) except that she was also observed occasionally in a contained backyard. In contrast, the apes were observed in their home, but also while exploring the surrounding forest. This forest contained many paths, landmarks, and potential destinations. Each destination contained a particular type of treat, such as a specific food that was often not available when foraging on other paths. The child in our study was therefore much more likely to encounter the same referents across multiple observations than were the ape participants. In order for reference to be observed transferring across modalities, future cross-species comparisons might benefit by constraining the number of possible referents and ensuring that similar referents are available across multiple sessions.

It is also important to note that the distinction between gestures and symbols made for the purposes of the current study is somewhat arbitrary (see Kendon, 2000; Capirci and Volterra, 2008; for a discussion of this). Notably, use of the lexigram board necessarily involves gesture. Gestures also continue to play a role in human communication across development, although their role and the frequency with which different gestures are used changes (Capirci and Volterra, 2008). Rather than being supplanted by speech, gesture may have co-evolved with speech (Corballis, 2002).

### Species differences and similarities in evidence of communicative intent

It is important to note that all three species of the clade used gesture communicatively and that all three species exhibited the same set of markers of communicative intent: eye gaze, vocalization, and persistence. Cladistic analysis suggests that these markers of communicative intent in the gestural modality were present in our common ancestor five to six million years ago. The combination of gesture and vocalization may have particular importance in language origins (Cartmill and Maestripieri, 2012) – given the existence of gesture-speech synchrony in human adults (McNeill, 1992) and gesture-vocalization synchrony in 2- and 3-months-old human infants (Fogel and Hannan, 1985). Future research should determine if other measures of communicative intent that were not assessed in the current study, such as tactile contact, occur equally frequently across species.

In line with our hypothesis, the human child more frequently paired gestures with vocalization than the apes. This association of gesture and vocalization, as well as the existence of gestures unique to the child (e.g., nodding, waving), constitute additional evidence suggesting the co-evolution of gesture and speech after the evolutionary divergence of the hominid line five to six million years ago.

Contrary to our hypothesis, and to previous comparisons of older apes to human children (Leavens and Hopkins, 1999), the human child more frequently paired eye contact with gestures than the apes did. However, closer analysis showed that this was because the child more frequently accompanied a single gesture with more than one marker of communicative intent than the apes did. This multimodal expression of communicative intent, normative for the child but less common for the apes, suggests strengthening of the use of multiple modalities to express communicative intent after the divergence of the hominid line.

### Species differences in types of gestures

The human child produced a far greater number of pointing gestures than did the apes; in contrast, the apes produced a greater number of reaching gestures than did the child. Only the human child produced showing gestures. Together these findings provide gestural evidence that ape communication is more instrumental than that of human children; in contrast, children gear their communication more to the sharing of experience with another (Tomasello, 2006). These gestural findings concerning pointing and reaching replicate the species-comparative pattern found on the symbolic level (Lyn et al., 2011).

However, we must not forget that both pointing and reaching were present in all species, in the same way that both declarative and imperative symbol productions are present later in development of the same apes, as well as two human children (Lyn et al., 2011). Thus, across the clade, the development of symbols builds on the pattern of communicative functions that are present earlier in ontogeny in the gestural mode. As with markers of communicative intent, the array of gesture types, likely present in the clade’s common ancestry, provided the building blocks upon which natural selection could work, making more adaptive traits more frequent as phylogenetic development proceeded – perhaps increasing the relative frequency of declaration in the human line as an enhanced stepping stone to human language. We can even conceptualize showing, unique to the human child, as a further evolutionary development growing out of declarative pointing as its ontogenetic foundation (Camaioni, 1997).

The finding that the human child pointed more relative to reaching than the apes is again contrary to previous comparisons of older language-enculturated apes and human infants (Leavens and Hopkins, 1999). Differences between the current findings and those with older language-enculturated apes suggest that it is important to take developmental stage into consideration when comparing across species and that changes in the frequency of pointing and eye contact may emerge across development among language-enculturated apes. The importance of taking a life-span approach to cross-species comparisons of symbolic development should not be underestimated.

### Limitations

When comparing development across three species, it is difficult to equate the species in terms of developmental level as skills are likely to develop at variable rates across species. Having more representatives of each species could increase our understanding of normative measures of development in each species and allow us to compare developmental stages across species more effectively. Additionally, a greater number of representatives of each species would allow us to disentangle species and individual differences.

Reliability between coders was substantially higher for some of the coding decisions (particularly for type of communication and for whether or not it was communicative) for the human child than it was for the apes. Nonetheless, inter-rater reliability reached acceptable standards for every species. These differences in coding reliability between the human child and the apes could be due to poorer video quality for the ape data and to difficulty on the part of human coders in coding ape gestures.

While lexigrams share a number of important similarities with words, such as an arbitrary correspondence between symbol and referent, they also have key differences. For example, ideas expressed in lexigrams do not always have a one-to-one correspondence to ideas expressed with words. For example, the lexigram “Sue’s-gate” is a single lexigram that could mean either a landmark or a more complex relation between a gate and a person. However, given that the semantic complexity of symbols was not our object of study, this difference should not have affected our results.

A more important difference between lexigrams and words is that lexigrams could be coded only when interpreted by a human caregiver or glossed by a machine while human speech needed only to be responded to in order to be coded. We did not code the number of lexigrams that were neither interpreted nor glossed and thus excluded from analyses. However, given that each ape was typically paired with a single caregiver who was intent on encouraging and capturing all of the ape’s communicative attempts, it is likely that only a small proportion of the apes’ lexigram use went unrecorded in the current analyses. In any case, it is likely that a small proportion of the child’s verbal communications were not responded to, so it is possible that there was no difference in selectivity between ape and child communication.

### Future directions

Future research should also examine the emergence of imperative (requests for something to be granted) or declarative (attempts to cause another to see what one sees: Bates et al., 1975) gestures across species. Although indication for declarative purposes is rarely reported in apes, it is more common and varied for language-enculturated apes (Greenfield and Savage-Rumbaugh, 1990; Brakke and Savage-Rumbaugh, 1996; Krause, 1997; Lyn et al., 2011).

In order to better investigate ontogenetic and phylogenetic relations between gesture and speech, future cross-species comparisons should distinguish between dyadic and triadic gestures as well as between deictic, iconic (or picture-like), and representational gestures. Dyadic gestures, referring to another, may be more developed in apes than triadic gestures, referring to objects. Even within triadic gestures, it is possible that apes use them to refer to other living beings more, while children in industrial societies use them to refer more to inanimate objects.

With respect to action gestures, Tanner and Byrne (1996) suggested that the ancestors of humanity probably communicated through iconic gestures about actions rather than objects because apes often focus on actions when gesturing. Indeed, comparisons of sign language produced by two deaf children and two language-trained gorillas demonstrated that while their early lexicons were similar in composition, more of the gorillas’ first signs depicted iconic actions than the children’s (Bonvillian and Patterson, 1993). It would be intriguing to examine developmental changes in types of gestures across species in order to investigate the relative importance of iconic relative to deictic gestures for symbolic development. Future research should also examine the relative frequency of imitated gestures and gestures denoting objects, actions, or other living beings across species in order to evaluate the theory that imitative gestures, or gestures denoting actions, may have been particularly important for language development (Rizzolatti and Arbib, 1998).

## Conclusion

What does this study tell us about the relationship between symbols and gestures? It provides evidence of a phylogenetic and ontogenetic transition from gesture to symbol. At the same time, it provides new evidence for the co-evolution of gesture and speech. The study documents clear similarities and differences in the ontogeny of communication of a chimpanzee, a bonobo, and a human child. The similarities provide insights into shared potential which could have helped our ancestors develop language from gesture. Differences suggest ways that humans may have diverged from other members of the clade in their communicative development, and provide evidence for the co-evolution of gesture and speech.

## Conflict of Interest Statement

The authors declare that the research was conducted in the absence of any commercial or financial relationships that could be construed as a potential conflict of interest.
